# Characterization of subcutaneous and omental adipose tissue in patients with obesity and with different degrees of glucose impairment

**DOI:** 10.1038/s41598-019-47719-y

**Published:** 2019-08-05

**Authors:** Anna Belligoli, Chiara Compagnin, Marta Sanna, Francesca Favaretto, Roberto Fabris, Luca Busetto, Mirto Foletto, Chiara Dal Prà, Roberto Serra, Luca Prevedello, Chiara Da Re, Romeo Bardini, Claudia Mescoli, Massimo Rugge, Paola Fioretto, Scilla Conci, Silvia Bettini, Gabriella Milan, Roberto Vettor

**Affiliations:** 1 Department of Medicine, University of Padua, Internal Medicine 3, 35128 Padua, Italy; 2 Center for the Study and the Integrated Treatment of Obesity, Padua Hospital, 35128 Padua, Italy; 3 Department of Surgical, Oncological and Gastroenterological Sciences, University of Padua, Division of General Surgery, 35128 Padua, Italy; 4 Department of Medicine, University of Padua, Surgical Pathology and Cytopathology Unit, 35121 Padua, Italy

**Keywords:** Stem-cell niche, Obesity, Diabetes

## Abstract

Although obesity represents a risk factor for the development of type 2 diabetes mellitus (T2DM), the link between these pathological conditions is not so clear. The manner in which the different elements of adipose tissue (AT) interplay in order to grow has been suggested to have a role in the genesis of metabolic complications, but this has not yet been fully addressed in humans. Through IHC, transmission electron microscopy, cytometry, and *in vitro* cultures, we described the morphological and functional changes of subcutaneous and visceral AT (SAT and VAT) in normoglycemic, prediabetic and T2DM patients with obesity compared to lean subjects. In both SAT and VAT we measured a hypertrophic and hyperplastic expansion, causing similar vascular rarefaction in obese patients with different degrees of metabolic complications. Capillaries display dysfunctional basement membrane thickening only in T2DM patients evidencing VAT as a new target of T2DM microangiopathy. The largest increase in adipocyte size and decrease in adipose stem cell number and adipogenic potential occur both in T2DM and in prediabetes. We showed that SAT and VAT remodeling with stemness deficit is associated with early glucose metabolism impairment suggesting the benefit of an AT-target therapy controlling hypertrophy and hyperplasia already in prediabetic obese patients.

## Introduction

Obesity and type 2 diabetes mellitus (T2DM) are closely linked global health care problems. Although the close relationship between weight gain and development of T2DM is well established, factors determining or accelerating the progression from the former to the latter continue to be debated^1^. The microvasculature seems to play a central role in glucose homeostasis; impaired capillary recruitment and tissue perfusion have been shown to reduce glucose uptake, leading to insulin resistance (IR) and metabolic alterations^2^. It has been demonstrated that low vascular density in adipose tissue (AT) is associated with an abnormal metabolic profile^3–5^. Furthermore, microangiopathy is a hallmark of diabetes complications. The cellular elements of the microvasculature appear to be particularly sensitive to injury from sustained hyperglycemia. The microvascular impairment or damage differs considerably between different tissues and organs, but it is possible to assume that all organs are affected simultaneously to a greater or lesser degree^6^. Moreover, there is now abundant evidence that microvascular dysfunction/disease is not restricted to the presence of Type 1 or 2 diabetes but is also present in many diseases which are, or represent, the first step in the progression toward diabetes itself, such as obesity. Therefore, we supposed that capillary density decrease and microangiopathy appear in AT with the worsening of glucose impairment; to this end, we examined AT vascularization in three groups of BMI-matched obese patients with different glycemic profiles, comparing them with non-diabetic normal weight subjects.

On the other hand, the existence of a metabolically protected but obese phenotype supports the paradox of the expandability of healthy AT^7^. In adulthood, AT, through the concomitant expansion of its capillary network, grows by hypertrophy and hyperplasia^8,9^. Thus, changes in adipocyte morphology and in adipose stem cells (ASCs) number and phenotype could play a key role in the metabolic complication development in obesity. Moreover, although ASCs, which are responsible for hyperplastic growth, have been investigated during recent years^10^, their origin and relationship with endothelial progenitors remain burning issues^11^.

Since human visceral AT (VAT) is less accessible than subcutaneous AT (SAT) our knowledge of the structural and functional features of this depot are still limited^12^, we studied both depots to assess whether they had similar features and roles in the presence of glucose impairment.

Considering different aspects of AT morphology, cell biology and the microvascular network we aimed to gain further knowledge about the role of AT remodeling as a function of the metabolic complication in obesity.

## Results

### Patients’ Clinical Characterization

The demographic, anthropometric and metabolic characteristics of the lean subjects and obese patients are detailed in Table 1. The lean subjects, who were age-matched with the obese patients, showed a lower incidence of comorbidities (Hypertension, Dyslipidemia, Obstructive Sleep Apnoea Syndrome). As expected, fasting plasma glucose (FPG) was similar in the lean and obese normoglycemic (ob N) patients; BMI and blood leptin level were not statistically different in the three groups of obese patients. FPG levels and the HOMA_IR_ were statistically different in the obese groups; they rose markedly as glycemic impairment increased (obese prediabetic and diabetic patients, ob preDM and T2DM). Ob T2DM patients displayed a mean glycated hemoglobin A1c (HbA1c) value of 66 ± 21 mmol/mol (8.2 ± 4.1%) and 56% of them presented microalbuminuria (181 ± 186 mg/g creatinine) (data not shown). Regarding the three most important microvascular complications (nephropathy, neuropathy and retinopathy) only 11/50 patients reported one or more of them (data from 7 patients were not available). This low rate of diabetic microvascular complications is probably due to the short duration of the diabetes. Indeed 58% (33/57) of our patients had had a recent disease diagnosis (less than 5 years). Thirty-eight percent of the ob preDM patients had impaired fasting glucose, 26% had impaired glucose tolerance, 36% had both. The glucose and insulin area under the curve (AUC) were thus lower in the ob N compared to the ob preDM patients. The incidence of comorbidities and the systemic inflammation markers (high sensitivity C-reactive protein, hs-CRP; tumor necrosis factor-alpha, TNF-α; interleukin-6, IL-6) were higher in the ob T2DM compared to the ob N patients.

**Table 1:** Characteristics and Clinical Parameters of the Lean Subjects and Obese Patients.

	Patient Groups				*P* value					
	lean (n = 18)	ob N (n = 62)	ob preDM (n = 58)	ob T2DM (n = 57)	lean vs. ob N	lean vs. ob preDM	lean vs. ob T2DM	ob N vs. ob preDM	ob N vs. ob T2DM	ob preDM vs. ob T2DM
Female sex-n°(%)	11(61.1)	50(80.6)	45(77.6)	32(56.1)	0.12	0.22	0.92	0.85	0.007	0.02
Age (y)	48 ± 12	41 ± 9	45 ± 10	52 ± 9	0.06	0.26	0.29	0.16	<0.001	0.001
Hypertension-n°(%)	2(11.1)	24(38.7)	31(53.4)	53(92.9)	0.06	0.004	<0.001	0.15	<0.001	<0.001
Dyslipidemia- n°(%)	1(5.5)	33(53.2)	32(55.2)	45(78.9)	<0.001	<0.001	<0.001	0.98	0.006	0.01
OSAS- n°(%)	0(0)	8(12.9)	16(27.6)	25(43.8)	0.19	0.009	0.002	0.07	<0.001	0.10
BMI (kg/m^2^) *	24 ± 2	46 ± 8	47 ± 7	48 ± 8	<0.001	<0.001	<0.001	0.83	0.10	1.0
FPG (mmol/L)	5.1 ± 0.7	5.0 ± 0.3	5.9 ± 0.5	9.4 ± 4.0	1.0	0.006	<0.001	<0.001	<0.001	<0.001
Insulin (mU/L)	—	16 ± 10	26 ± 20	33 ± 32	—	—	—	0.003	<0.001	0.43
HOMA_IR_ †	—	3.7 ± 2.4	6.9 ± 5.7	12.3 ± 12.5	—	—	—	<0.001	<0.001	0.03
Glucose-AUC (mmol/L) min ‡	—	1165 ± 125	1490 ± 171	—	—	—	—	<0.001	—	—
Insulin-AUC (mU/L) min ‡	—	13113 ± 6989	17858 ± 9843	—	—	—	—	0.007	—	—
Waist (cm) §	—	127 ± 14	132 ± 11	137 ± 16	—	—	—	0.04	<0.001	0.48
Total Cholesterol (mg/dL)	—	188 ± 30	192 ± 37	185 ± 39	—	—	—	0.59	0.59	0.59
HDL-Cholesterol (mg/dL)	—	48 ± 11	46 ± 13	43 ± 11	—	—	—	0.60	0.02	0.50
LDL-Cholesterol (mg/dL)	—	119 ± 25	120 ± 36	113 ± 35	—	—	—	0.55	0.55	0.55
Triglycerides (mg/dL)	—	110 ± 47	137 ± 79	163 ± 81	—	—	—	0.23	<0.001	0.06
hs-CRP (mg/L)	—	5.1 ± 3.3	6.9 ± 3.2	7.6 ± 3.4	—	—	—	0.02	0.001	1.0
TNF-α (ng/L)	—	7.6 ± 2.9	7.7 ± 2.9	9.1 ± 3.2	—	—	—	1.0	0.01	0.02
IL-6 (ng/L)	—	3.4 ± 2.5	3.2 ± 1.3	4.4 ± 2.4	—	—	—	0.81	0.003	0.08
Leptin (µg/L)	—	39 ± 15	39 ± 16	36 ± 15	—	—	—	0.47	0.47	0.47

Clinical data of control subjects (lean), normoglycemic (ob N), prediabetic (ob preDM) and diabetic (ob T2DM) obese patients are presented as mean values ± standard deviation (SD). Statistical analysis was based on the One Way ANOVA test (age, total cholesterol, leptin) or a non-parametric Kruskal-Wallis test (BMI, FPG, waist, insulin, HOMA_IR_, HDL- and LDL-cholesterol, triglycerides, hs-CRP, TNF-α, IL-6) followed, when statistically significant by, respectively, the Holm-Sidak test or Dunn’s multiple comparison post-test. The Wilcoxon Mann Whitney test was used to compare glucose- and insulin-AUC values. Pearson’s chi-square test or Fisher’s exact test were used to compare dichotomous variables between groups.

^*^BMI was calculated by dividing weight in kilograms by the square of height in meters. ^†^HOMA_IR_ was calculated according to the following formula: [fasting serum insulin (mU/L) x fasting plasma glucose (mmol/L)]/22.5. ^‡^AUC was calculated using the trapezoidal method. ^§^Waist circumference was measured with a tailor-meter at the midpoint of the line connecting the inferior border of the lowest rib with the superior border of the iliac crest.

Clinical data of subgroups of subjects analyzed in each different experiment were reported in Supplementary Information (Supplementary Tables S1–S6).

### Adipose tissue morphological and gene expression studies

**Figure 1:**
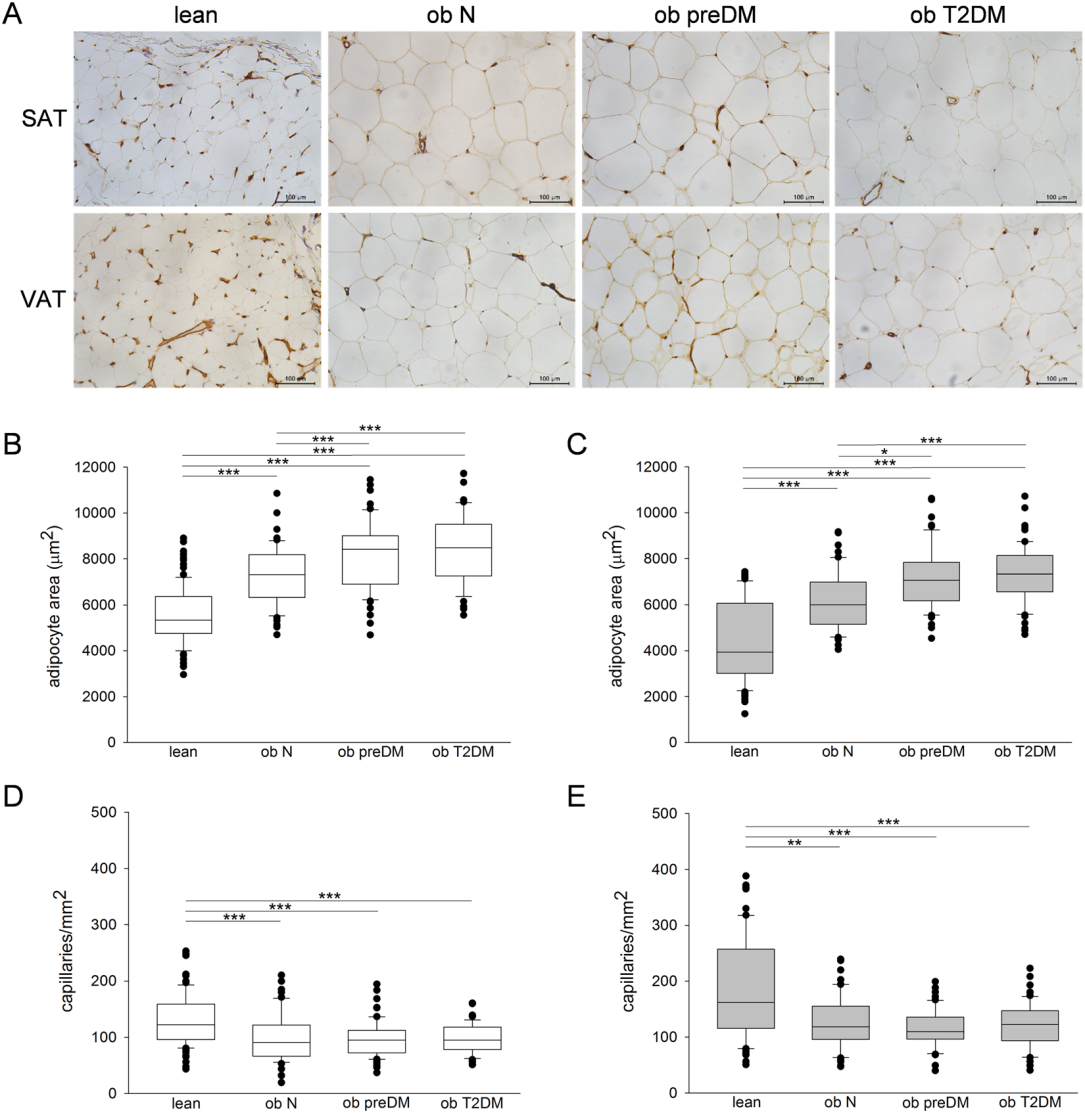
Immunohistochemistry for CD31, adipocyte size and capillary density analysis in human SAT and VAT. Representative photomicrographs of SAT and VAT sections from the 4 groups of patients studied: lean control subjects, normoglycemic (ob N), prediabetic (ob preDM) and diabetic (ob T2DM) obese patients stained with anti-CD31 antibody (**A**). Measurements of adipocyte area (µm^2^) and quantification of capillary density (number of capillaries per mm^2^) in the SAT (white boxes, **B**,**D**) and VAT (grey boxes, **C**,**E**) of 10 lean, 5 ob N, 5 ob preDM and 5 ob T2DM patients. The results are displayed as box plot graphs: the box represents the lower and upper quartiles, the line in the box represents the median, the whiskers show the lowest and highest values and the outliers indicating the median value of measurements for one field are represented by black circles. Data was analyzed using One Way ANOVA (Fisher LSD method) in (**B**) and Kruskal-Wallis test (Dunn’s method) in (**C**–**E**), *P ≤ 0.05, **P ≤ 0.01, ***P ≤ 0.001.

For the immunohistochemical (IHC) analysis, we selected patients with similar age among the groups described in Table 1. Histomorphometric evaluations (Fig. 1) highlighted that the SAT adipocytes were larger than the VAT ones in all the groups studied (*P* < 0.001, Mann-Whitney test). The median adipocyte area in both the SAT and VAT of all the obese subgroups was significantly higher compared to that of the lean group. Interestingly, adipocytes of ob preDM were larger than those of ob N and the ob T2DM subgroup showed an adipocyte size comparable to ob preDM (Fig. 1A–C). This is an important result, considering that the ob preDM patients selected for IHC analysis were “early” prediabetic. Indeed even if their glucose AUC was similar to the whole prediabetic population, their insulin AUC and HOMA_IR_ values were more similar to those of the ob N subgroup (ob preDM *vs* ob N IHC; HOMA_IR_: 3.6 ± 1.8 *vs* 3.3 ± 1.9, *P* = 1.0; insulin AUC: 15463 ± 12065 *vs* 10535 ± 4853 mU/L per min, *P* = 0.84) (Supplementary Table S1).

**Figure 2:**
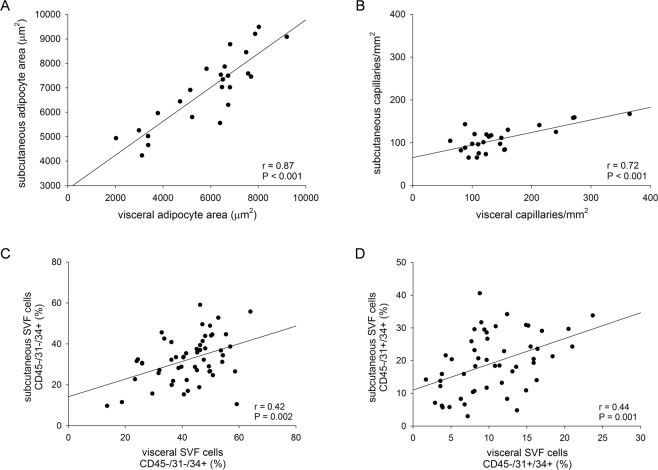
The correlation between data obtained from AT depots. The quantifications of adipocyte area (**A**), capillary density (**B**), adipose stem cells CD45−/31−/34+ (**C**) and endothelial progenitors CD45−/31+/34+ (**D**) in SAT and VAT depots were correlated using Pearson’s coefficient. Number of the patients analyzed was 25 in (**A**,**B**), 54 in (**C**) and 51 in (**D**).

Capillary density was higher in the VAT than in the SAT in all of the groups (*P* < 0.001, Mann-Whitney test) (Fig. 1D,E). The density of vessels was lower in both fat depots in the obese patients compared to the lean, but there were no differences between the obese subgroups (Fig. 1A,D,E). IHC data obtained from SAT and VAT positively correlate (Fig. 2A,B). Moreover, peroxisome proliferator-activated receptor-gamma isoform 2 (*PPARG2*) mRNA was significantly upregulated in the obese patients compared to the lean, and the ob T2DM group had the lowest levels of all three groups (Supplementary Fig. S1A,B).

Hypoxia inducible factor 1 alpha subunit (*HIF1A*) was upregulated only in the SAT of the obese patients compared to that of the lean group; the ob T2DM subgroup exhibited the highest expression level in SAT (Supplementary Fig. S1C,D). The vascular endothelial growth factor A (*VEGFA*) expression in the SAT and VAT was similar in all the patients analyzed (Supplementary Fig. S1E,F).

### Capillary basement membrane (CBM) analysis of adipose tissue

**Figure 3:**
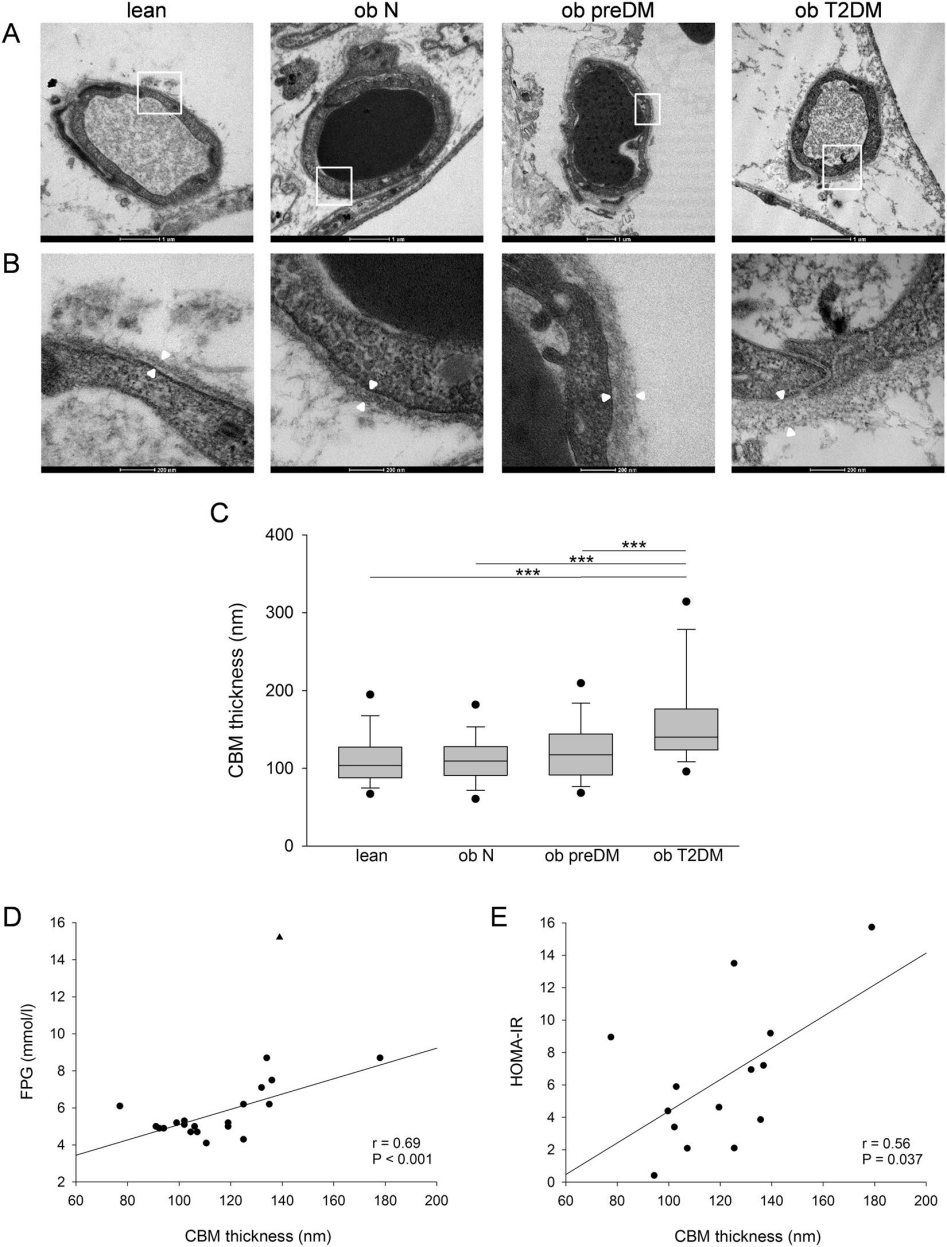
Capillary morphology, CBM thickness and correlation analysis in human VAT. Representative transmission electron micrographs of transverse sections of VAT capillaries in the lean control subjects and in the normoglycemic (ob N), prediabetic (ob preDM) and diabetic (ob T2DM) obese patients (**A**). Regions in the white boxes in (**A**) are shown at higher magnification in (**B**). White arrowheads indicate the CBM thickness, which was measured at multiple points in each micrograph. CBM thickness (nm) in the VAT was analyzed in 6 lean, 5 ob N, 5 ob preDM and 5 ob T2DM subjects (**C**). Data are reported as box plot graph with the lowest and highest values (whiskers), the medians (lines) and the 5^th^ and 95^th^ percentiles (black circles outside the whiskers). Correlation analysis was performed between CBM thickness, FPG of all patients with the exclusion of one outlier indicated by a triangle, and HOMA_IR_ of 14 obese patients (**D**,**E**). Data was analyzed using the Kruskal-Wallis test (Dunn’s method) in (**C**), ***P ≤ 0.001, and Pearson’s correlation coefficient in (**D**,**E**).

In literature, CBM thickness is described as being correlated with age^13^, therefore in order to avoid this bias we selected the more possible age-matched patients to perform the transmission electron microscopy (TEM) analysis (Supplementary Table S2). It is worth noting that no patients in the ob T2DM group selected for TEM analysis reported diabetic microvascular complications but 60% presented microalbuminuria. The CBM thickness of the VAT of these patients was measured to investigate AT microangiopathy (Fig. 3). Only the ob T2DM patients showed a significant increase in CBM thickness compared to the other 3 groups (Fig. 3C). No significant differences were observed between the lean, ob N and preDM patients, although a pattern of progressive thickening was noted.

The CBM thickness was strongly positively correlated with FPG (Fig. 3D) and HOMA_IR_ (Fig. 3E) and not with age (data not shown).

### The immunophenotype and adipogenic potential of stromal vascular fraction (SVF)

**Figure 4:**
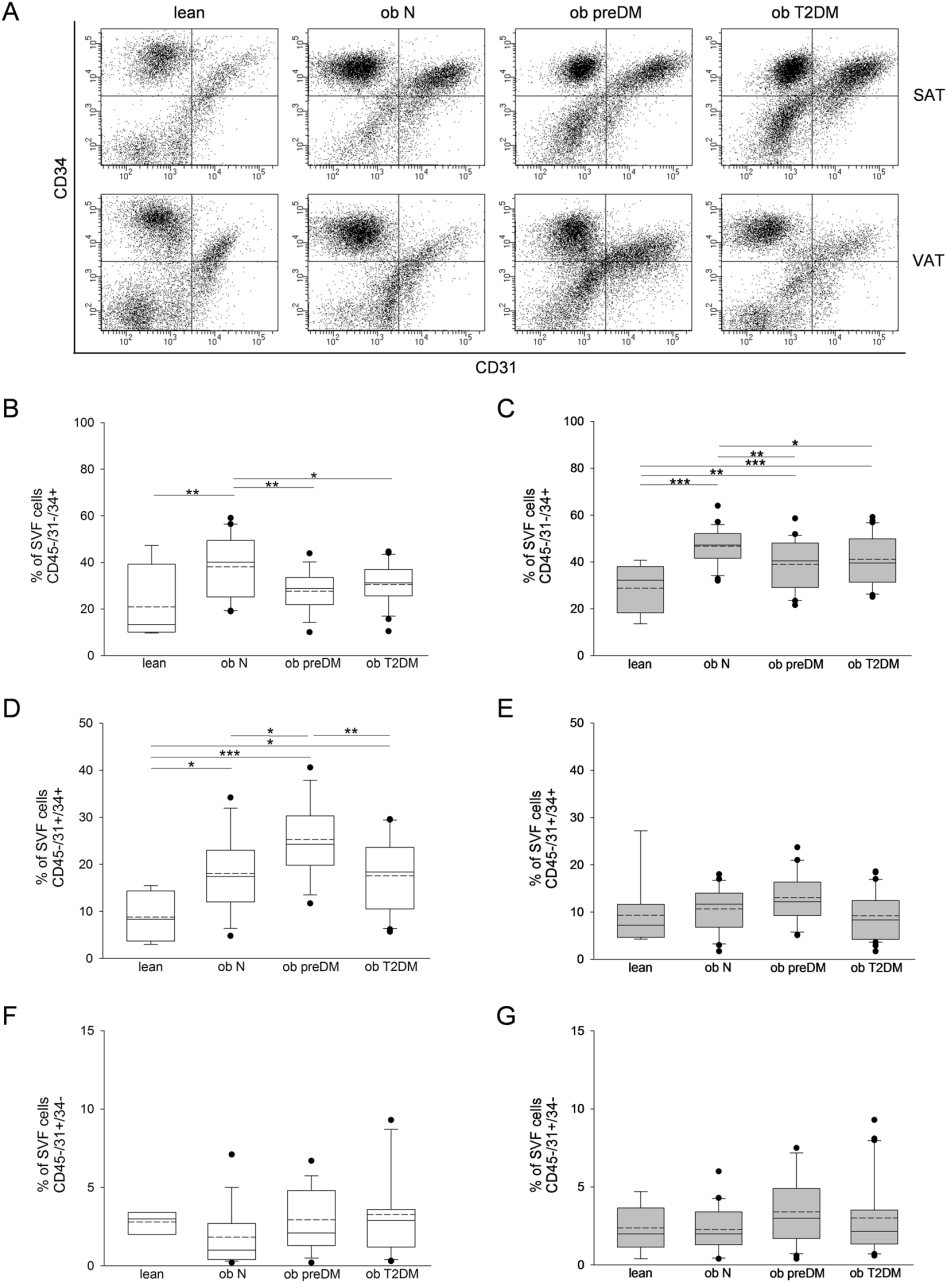
Flow cytometric analysis of SVF cells obtained from human SAT and VAT. Representative flow cytometric dot plots of surface markers CD34 *vs* CD31 determining the percentage of adipose stem cells (ASCs) (CD45−/31−/34+), endothelial progenitor cells (CD45/31+/34+) and endothelial mature cells (CD45−/31+/34−) within SVF freshly isolated from SAT and VAT of the lean control subjects and normoglycemic (ob N), prediabetic (ob preDM) and diabetic (ob T2DM) obese patients (**A**). Quantification of ASCs (**B**,**C**), endothelial progenitor (**D**,**E**) and endothelial mature cells (**F**,**G**) contained in SVF from SAT (white boxes, **B,D,F**) and VAT (grey boxes, **C,E,G**). The percentage of cells were displayed as box plot graphs with the lowest and highest values (whiskers), the medians (lines), the means (dashed lines) and the outliers (black circles). The number of AT samples analyzed for each group was: 4 lean, 20 ob N, 15 ob preDM, 23 ob T2DM in SAT and 9 lean, 23 ob N, 21 ob preDM, 30 ob T2DM in VAT. The data was analyzed using the One Way ANOVA test followed, when statistically significant, by Fisher LSD test in (**B**–**D**) and Kruskal-Wallis test (Dunn’s method) in (**E**–**G**), *P ≤ 0.05, **P ≤ 0.01, ***P ≤ 0.001.

The percentage of SAT-ASCs (CD45−/31−/34+) on the SVFs was significantly higher in the ob N compared to that in the lean group (Fig. 4A,B). VAT (Fig. 4C) in all the obese patients showed a significant ASC enrichment compared to that in the controls. Both SAT- and VAT-ASCs percentage was lower in the ob preDM compared to the ob N subgroup; this reduction was observed also in the ob T2DM subgroup to the same extent (Fig. 4B,C). The VAT contained a higher number of ASCs than the SAT did in all the patients. The number of adipogenic precursors was not correlated with age (data not shown), though the ob T2DM patients analyzed were older than the ob N and preDM patients (Supplementary Tables S3, S4).

The ASC phenotype was further characterized in the attempt to evaluate the expression of typical mesenchymal stem cell markers such as CD73, CD90, CD105, CD271 and pericyte marker CD146 (Supplementary Table S7). More than 90% of the CD45−/31−/34+ cells expressed CD73 and CD90 in both depots and similar patterns occurred in all the patient subgroups. CD105 was, instead, expressed only by 30–70% of the CD45−/31−/34+ in both the SAT and VAT of the lean subjects and obese patients. More specifically, the percentage of CD45−/31−/34+/105+ cells was similar in the SAT of all the groups, but it had increased in the VAT of all the obese subgroups compared to the lean group. Similar results were obtained with regard to CD271 antigen expression. A small ASC percentage (<2%) expressed the pericyte marker CD146 and there were no differences between the patient groups or AT depots.

The percentage of endothelial progenitors (CD45−/31+/34+) was higher in the SAT of all the obese patients compared to that in the lean (Fig. 4D) and this trend was similar, even if not significant, in the VAT (Fig. 4E). Importantly, the percentage of stem cells in the SAT was positively correlated with that in the VAT (Fig. 2C,D). No differences in the number of endothelial mature cells (CD45−/31+/34−) were observed in the two AT depots of the various groups (Fig. 4F,G).

**Figure 5:**
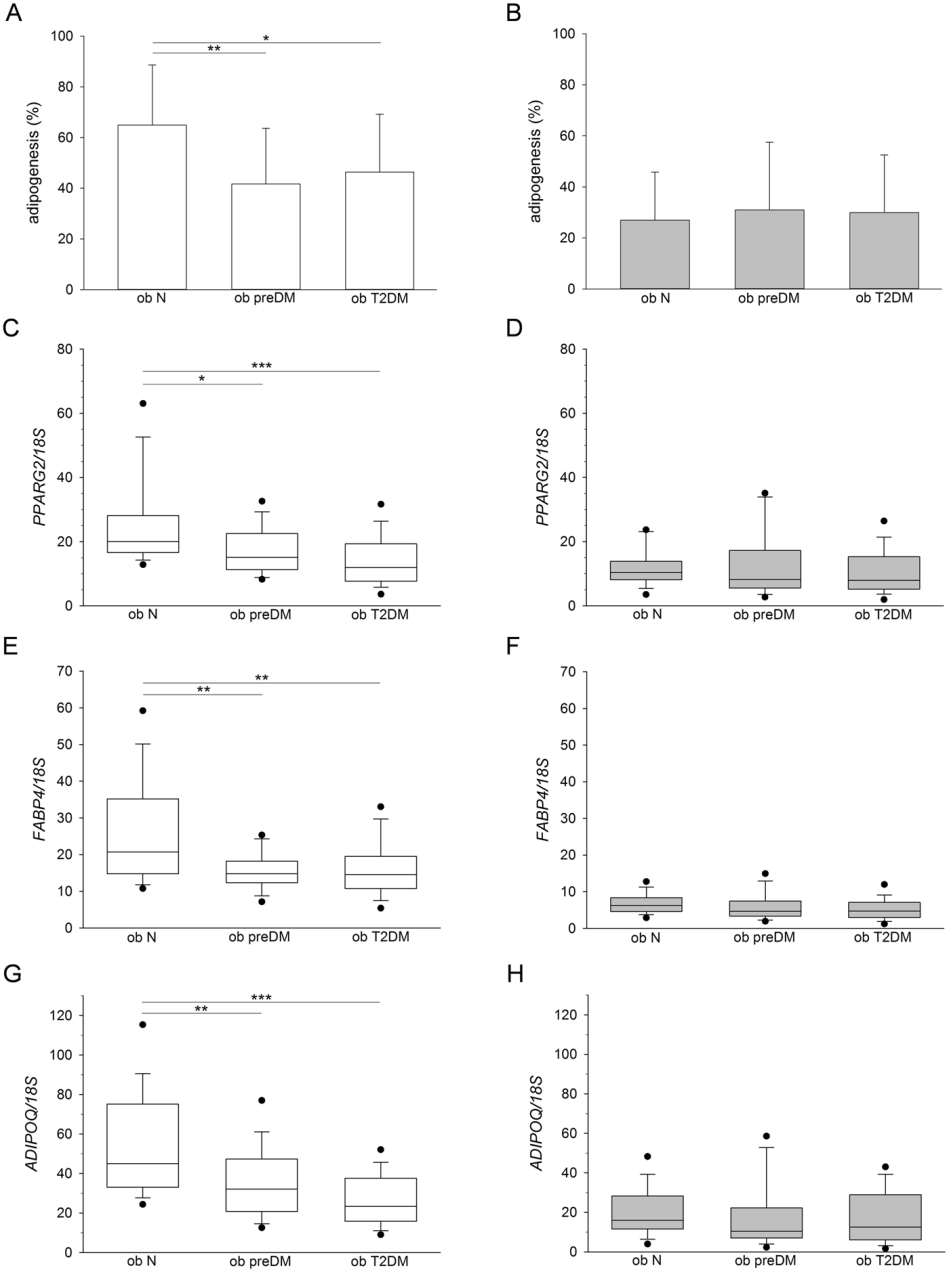
*In vitro* adipogenic potential of SVF cells obtained from human SAT and VAT. The percentage of mature adipocytes was obtained by optical microscope analysis, upon Oil-Red O staining, of *in vitro* adipogenic differentiated cell cultures isolated from SAT (white columns) and VAT (grey columns) of normoglycemic (ob N), prediabetic (ob preDM) and diabetic (ob T2DM) obese patients (**A**,**B**). The data, described as mean value ± SD, was analyzed using the One Way ANOVA test followed, when statistically significant, by Fisher LSD test in (**A**) and Kruskal-Wallis test (Dunn’s method) in (**B**), *P ≤ 0.05, **P ≤ 0.01. The patients analyzed were: 18 ob N, 15 ob preDM, 13 ob T2DM in SAT and 20 ob N, 25 ob preDM, 23 ob T2DM in VAT. *PPARG2* (**C**,**D**), *FABP4* (**E**,**F**), *ADIPOQ* (**G**,**H**) mRNA levels quantified in adipogenic differentiated cell cultures obtained from SAT (white boxes, **C,E,G**) and VAT (grey boxes, **D,F,H**) of ob N, ob preDM and ob T2DM patients, normalized to *18S* rRNA content, are reported as box plot graph with the lowest and highest values (wiskers), the medians (lines) and 5^th^ and 95^th^ percentiles (black circles outside the whiskers). Statistical analysis was performed using Kruskal-Wallis test followed by Dunn’s multiple comparison post-hoc test in (**C**–**H**), *P ≤ 0.05, **P ≤ 0.01, ***P ≤ 0.001. The patients analyzed were: 17 ob N, 15 ob preDM, 12 ob T2DM in SAT and 20 ob N, 25 ob preDM, 23 ob T2DM in VAT.

Lastly, the capacity to differentiate towards the adipogenic lineage was investigated in obese patients whose clinical characteristics are described in Supplementary Tables S5, S6. *In vitro* differentiation revealed a lower percentage of mature adipocytes from the SAT-SVF of ob preDM and T2DM compared to the ob N subgroup (Fig. 5A). SVFs isolated from the VAT displayed a very low adipogenic potential with no differences between the subgroups (Fig. 5B). These results were confirmed by the gene expression analysis of 3 adipose-specific genes (*PPARG2*, *FABP4* and *ADIPOQ*), quantified in cell cultures upon *in vitro* adipogenic differentiation (Fig. 5C–H).

## Discussion

Since AT plasticity contributes to the pathogenesis of obesity and related metabolic complications^9,14^, we aimed to explore AT remodeling in normoglycemic, prediabetic and diabetic patients with obesity.

Several lines of evidence suggested that AT vascularization changes could influence fat growth and its metabolic features^4,5,15,16^. Confirming and extending on previous studies, we showed the presence of capillary rarefaction in the SAT and VAT of the obese patients compared to the lean subjects^3,4,17,18^. Surprisingly, the degree of vascularization was similar in the obese subgroups independently of any further increase in adipocyte size or worsening in the metabolic profile (Fig. 1D,E). We demonstrated that among obese patients there are no differences in the number of AT capillaries, neither in VEGFA expression (Supplementary Fig. S1E,F) nor in the quantification of endothelial mature cells isolated from AT-SVFs (Fig. 4F,G). Thus, obesity-related T2DM did not seem to be triggered by vascular network defects. At the same time, the capillary rarefaction and the higher levels of the HIF1A we found in the SAT of the obese patients (Supplementary Fig. S1C) confirm the hypothesis that hypoxia could play a central role in AT dysfunction in obesity, as previously reported^3,18–22^. Our AT expression profile analysis showed that HIF1A upregulation is not associated with a parallel induction in VEGFA expression (Supplementary Fig. S1C,E). Although the endothelial precursors were higher in the AT of the obese patients compared to lean controls (Fig. 4D,E), they seemed to be unable to differentiate into mature endothelial cells or to form capillary structures (Figs 1D,E and 4F,G), suggesting that HIF1A upregulation during obesity induces only a detrimental transcriptional program contributing to AT dysfunction^17,19,20,23^.

Our TEM investigation in VAT highlighted a significant CBM thickening only in ob T2DM patients (Fig. 3A–C) and a positive correlation between this thickening and FPG and HOMA_IR_ (Fig. 3D,E), but not with BMI values. These results support the hypothesis that obesity per se does not affect CBM structure and that thickening is mostly related to the exposure to chronic hyperglycemia. Our results suggested that the capillaries of VAT of obese T2DM patients could be functionally impaired contributing to the worsening of hypoxic conditions and AT inflammation leading to a more severe adiposopathy associated with long-term metabolic complications present in overt T2DM. CBM thickening of the retina and the kidney has long been known to be a crucial anatomic feature of microangiopathy in patients with Type 1 and 2 DM^6^ but this is the first study describing VAT microangiopathy as a function of glucose metabolic impairment in obese patients.

Regarding AT morphology, we observed a clear increase in adipocyte size in patients with obesity when compared to lean controls at the same extension in SAT and in VAT, but intriguingly the largest cell size observed in patients with obesity and T2DM was also detected in the prediabetic condition (Fig. 1A–C). Thus, alterations in AT morphology did not seem to be a result of prolonged hyperglicemia, hyperinsulinemia or elevated IR degree in the patients studied. We recently published that the insulin signalling pathway is similarly active in the AT of prediabetic and normoglycemic obese patients and it is downregulated in diabetic obese patients^24^. Therefore, the increase in adipocyte size seems pre-existent to insulin signalling alterations and could be linked to genetic or epigenetic characteristics of patients. The increasing size of adipocytes in the obese patients compared to the lean controls and the general observation that the adipocytes were larger in the SAT than in VAT (Figs 1A–C and 2A) also concur with previous reports^25–27^. The huge AT expansion in obesity was also confirmed by the marked up-regulation of PPARG2 expression in both depots (Supplementary Fig. S1A,B).

We observed an increase of ASCs, which could be considered a marker of AT hyperplastic growth, in the obese patients compared to lean controls and a shrinking of their number in the obese patients with glycemic impairment (Fig. 4A–C). Accordingly, the *in vitro* adipogenic potential of SAT-SVF cells from the ob preDM and T2DM patients was lower than that in the ob N (Fig. 5A,C,E,G), supporting the theory that the balance between hypertrophy and hyperplasia in AT growth contributes to metabolic impairment^25,28–30^. Despite the fact we only had the possibility to perform cross-sectional observations on our obese cohort, several of our results were consistent with previous findings obtained by a prospective longitudinal study on the Pima Indian population^30^.

Although SAT seems to conserve some beneficial features such as the higher amount of endothelial precursors and a greater *in vitro* adipogenic potential, many of our findings obtained in SAT correlate with those obtained in VAT (Fig. 2) suggesting that both AT depots could play a role in severe obesity and metabolic complications.

**Figure 6:**
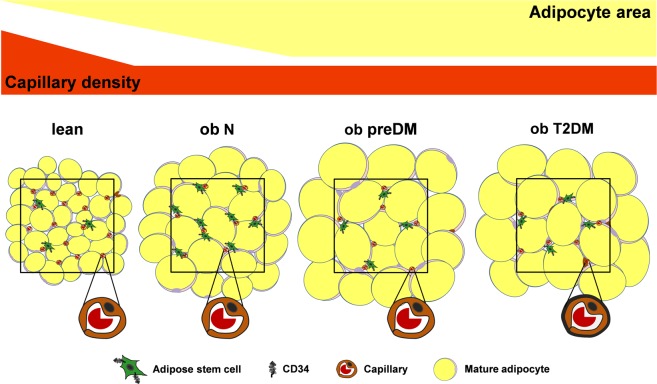
Adipose tissue remodeling in obesity and metabolic complications. During obesity AT grows both by hypertrophy and hyperplasia determining vascular rarefaction. Enhanced enlargement of mature adipocytes and a significant decrease in ASCs occur early in the prediabetic condition and these modifications are also present into overt diabetes, suggesting further expansion mainly by hypertrophy. AT capillaries display marked CBM thickening (black line) only in overt diabetes (AT microangiopathy), suggesting vascular dysfunction and further perfusion reduction. The black squares circumscribe 1 mm^2^. ob N = patients with obesity and normoglycemia; ob preDM = patients with obesity and prediabetes; ob T2DM = patients with obesity and diabetes.

Figure 6 well represents all our data showing that AT in obesity grows both through hypertrophy and hyperplasia causing vascular rarefaction that is not related to glucose metabolism impairment. Mature adipocytes are enlarged and there are significantly fewer ASCs early in the prediabetic condition. These alterations in AT remodeling are also present into overt diabetes in association with the development of AT diabetic microangiopathy.

Our results suggest that the dynamic balance between AT hyperplasia and hypertrophy, rather than vascular network impairment, is the early crucial alteration triggering the pathogenesis of impaired glucose homeostasis in severe obesity. Future studies will be necessary to identify the mechanisms leading to these AT structural changes and to develop a specific AT targeting therapy for prediabetic obese patients.

## Methods

### Patients

In order to investigate AT architecture, paired SAT and VAT samples were collected from 177 obese patients and from 18 normal weight non-diabetic (lean) subjects considered the control group. The baseline clinical evaluation included a complete medical history and clinical examination. The patients’ anthropometric measurements were taken, and hematological and biochemical parameters were determined (Table 1). The study’s exclusion criteria were: a history of malignancy, chronic inflammatory diseases such as ulcerative colitis and/or Crohn’s disease, active infectious diseases, drug or alcohol abuse and, for the lean subjects, T2DM.

All the obese patients, attending the Center for the Study and the Integrated Treatment of Obesity (Ce.S.I.T.O) of Padua Hospital between January 2014 and June 2016, were selected with a matched BMI. In accordance with the American Diabetes Association criteria they were divided into 3 groups depending on their glycemic profile: 62 were ob N, 58 ob preDM and 57 ob T2DM^31^. One hundred sixty-nine obese patients underwent a laparoscopic sleeve gastrectomy and 8 underwent a gastric bypass.

The lean subjects (18.5 ≤ BMI ≤ 24.9 Kg/m^2^) were attending the Division of General Surgery or the Ce.S.I.T.O of Padua Hospital; AT samples were harvested from them during abdominal surgery (laparoscopic cholecystectomy, fundoplication, colic resection for diverticular disease, rectal prolapse reduction).

All adipose tissue samples were collected during laparoscopic surgery in the abdominal region. In particular, VAT was obtained harvesting at least 1 cm^3^ of omental fat, while SAT was obtained excising 1 cm^3^ of subcutaneous fat at trocar site, both in obese and lean subjects.

The incidence of the three principal obesity-related comorbidities (hypertension, dyslipidemia and obstructive sleep apnoea syndrome) was calculated for each group of patients. Patients were considered affected by one of the above-mentioned comorbidities when they were receiving specific treatment or when they met international criteria^32–34^.

### Biochemical analysis

Blood biochemical analyses were performed after an 8-hour fast. FPG, insulin, lipid profile, hs-CRP, TNF-α, IL-6 and leptin levels were measured in all of the obese patients studied. The HOMA_IR_ was used to calculate the insulin resistance index^35^. A 3-hour 75 g oral glucose tolerance test (OGTT) was performed for blood glucose and insulin plasma levels at baseline and 30, 90, 120, 150, 180 minutes after glucose loading (180 mL of syrup with 82.5 g glucose monohydrate equal to 75 g of glucose). Glucose and insulin AUC was calculated. OGTT was not performed in obese patients with a history of diabetes, and fasting insulin was not measured in patients receiving insulin treatment, meaning that HOMA_IR_ was not calculated in these patient categories. In patients with T2DM we also measured HbA1c (by high performance liquid chromatography) and microalbuminuria by the albumin/creatinine ratio (urinary albumin/creatinine ratio <30 mg/g creatinine was considered normal). Biochemical measurements were performed using diagnostic kits standardized according to the World Health Organization First International Reference Standard: glucose (Glucose HK Gen.3, Roche Diagnostic, USA), insulin, IL-6, TNF-α (IMMULITE 2000 Immunoassay, Siemens Healthcare GmbH, Germany), hs-CRP (CardioPhase High Sensitivity C-Reactive Protein, Siemens Healthcare) and leptin (Leptin-RIA-CT, Mediagnost, Germany).

### IHC analysis

Paired SAT and VAT samples were fixed in 4% formaldehyde (Diapath S.p.A, Bergamo, Italy), paraffin embedded, cut into 5 µm thick sections and stained with monoclonal mouse anti-human CD31 (clone JC70A, 1:100; DakoCytomation, Carpinteria, CA, USA). The selected patients were of similar age, while their main demographic, anthropometric and metabolic characteristics were comparable to the whole population (Supplementary Table S1). Indirect immunohistochemistry was performed with a Dako labeled streptavidin biotin-horseradish peroxidase kit. AT images were captured at 20X magnification with a Leica DFGC450 digital camera (Leica DM LB2 light microscope) in at least 10 different fields (up to a minimum of 200 random adipocytes) per specimen of each patient. The adipocytes’ sizes were measured using LAS Software (Leica Microsystems Inc., Deerfield, IL, USA). The median adipocyte area for each field was used to calculate the median adipocyte area for each subgroup of patients. The number of capillaries per mm^2^ was determined counting positive capillary lumens in the same fields in which the adipocytes’ areas were measured.

### TEM analysis

VAT samples were fixed in 2% glutaraldehyde-2% paraformaldehyde in 0.1 M sodium cacodylate buffer pH 7.4 for 1 hour at 4 °C, postfixed in osmium tetroxide 1% for 1 hour at 4 °C and embedded in an Epon-Araldite mixture. Ultrathin sections (60–70 nm) were obtained with an Ultratome V ultramicrotome (LKB, Stockholm, Sweden), counterstained with uranyl acetate and lead citrate and viewed with a Tecnai G2 microscope (FEI, Hillsboro, OR, USA) operating at 100 KV. Images were captured with a Veleta (Olympus Soft Imaging System GmbH, Münster, Germany) digital camera.

Morphometric evaluation of CBM from TEM images at 93000X magnification was performed using *Image J* software, as described by Baum, on at least 45 capillaries for each group of patients^36^. Only images showing capillaries cut perpendicular to their long axes were used for analysis.

### SVF extraction and flow cytometry analysis

Depending on tissue availability, SVF cells from AT were freshly isolated for *ex vivo* multiparametric flow cytometric analysis and/or primary adipocytes culture.

SAT and VAT biopsies were minced and digested in collagenase type II solution (1 mg/mL) (Sigma-Aldrich, St. Louis, MO, USA), centrifuged (350 xg, 10 min) and the red blood cells were removed using a standard lysis buffer, as previously described by Sanna *et al*.^37^.

1 × 10^5^ SVF freshly isolated cells were washed with a cold FACS buffer (2% BSA/PBS) and simultaneously incubated in the dark for 10 min at room temperature with monoclonal mouse anti-human fluorochrome-conjugated antibodies (BD Biosciences) in different combinations showen in Supplementary Table S8. After washing with the FACS buffer, labeled cells were resuspended in 200 µl of FACS buffer and acquired (30000 events/sample) by a FACSCanto^TM^ Flow Cytometer (BD Biosciences). Data acquisition and analysis were performed using BD FACSDiva™ software. Regions and gates were set by a negative control, isotype-matched PE-IgG1, FITC-IgG1, APC-IgG1 and PerCP-Cy5.5-IgG1 monoclonal antibodies (BD Biosciences). ASCs, identified as CD45−/31−/34+, were quantified as percentages in the SVF morphological gate. The endothelial progenitor and mature cells were identified, respectively as CD45−/31+/34+ and CD45−/31+/34−, and quantified using the same procedure^11,38^. The expression of additional mesenchymal stromal/stem cells markers (CD73, CD90, CD105, CD271) and of pericyte marker CD146 were quantified in the ASCs’ immunological gate by subtracting the FITC signal (CD31-FITC and CD45-FITC).

### Adipogenic differentiation

1 × 10^5^ SVF cells per well were seeded in duplicate in 96-well plates in a human standard medium and, when the cells reached confluence, adipogenic differentiation was induced using an adipogenic medium as described in Borgo *et al*.^24^. At the end of differentiation (12 days), RNA was extracted from the cell cultures (two 96-well replicates lysed together), as described in the paragraph below, and the cell cultures (two 96-well replicates) were fixed in 10% formalin/PBS and stained with Oil-Red O (Sigma-Aldrich) solution in 40% isopropanol. After 3 PBS washes, the percentage of mature adipocytes was estimated by observing the specific staining for lipid droplets during double blind observation with a Leica DM IL LED inverted microscope.

### RNA extraction and real time PCR

Total RNA was extracted from paired SAT and VAT samples and from *in vitro* adipogenic differentiated cell cultures (12 days) using RNeasy Lipid Kits (Qiagen Inc., Valencia, CA, USA), following the supplier’s instructions, and quantified using NanoDrop technology (Fisher Scientific SAS, Illkirch Cedex, France). Five hundred ng of RNA was treated with DNase Treatment and Removal Reagents (Thermo Fisher Scientific Inc., Waltham, MA, USA) and reverse-transcribed for 1 h at 37 °C with 150 ng random hexamers, 0.5 mM dNTPs, 20 units of RNAsin Ribonuclease Inhibitor and 200 units of M-MLV RT (Promega, Madison, WI, USA). Oligonucleotide sequences and amplification conditions are shown in Supplementary Table S9. Real Time PCR was carried out with Platinum SYBR Green qPCR SuperMix-UDG (Thermo Fisher) on a DNA Engine Opticon 2 Continuous Fluorescence Detection System (MJ Research, Waltham, MA, USA). Duplicate 5 ng cDNA samples were normalized by *RNA18S* content.

### Statistical analysis

Mean values with standard deviations (SD) and percentages were used to describe the clinical parameters. All the variables were tested for normality using the Shapiro-Wilk test and, based on their distribution, the differences between the groups were evaluated using a parametric One Way ANOVA test or a non-parametric Kruskal-Wallis test; these were followed, respectively, by Fisher’s LSD/Holm-Sidak or by Dunn’s multiple comparison post-test. Pearson’s chi-squared test or Fisher’s exact test were used to compare dichotomous variables. Student’s t-test and the Mann-Whitney non parametric test were used when two groups were compared. All the P values were two-sided; a P value lower than 0.05 was considered statistically significant. Pearson’s correlation coefficient (r) and the relative P values were calculated to measure the strength of the statistical correlation between two variables. Statistical analyses were performed using STATISTICs Software (StatSoft 7.1).

### Study approval

The Padua Ethical Committee for Clinical Research approved the study involving patients confirming that all methods were performed in accordance with the relevant guidelines and regulations (2892 P approved 10/06/2013); each subject gave informed written consent for AT biopsies.

## Supplementary information

Characterization of subcutaneous and omental adipose tissue in patients with obesity and with different degrees of glucose impairment

## Data Availability

The authors declare that the data supporting the findings of this study are available within the article and its supplementary information files.
