# Supplementary material for: Characterization of subcutaneous and omental adipose tissue in patients with obesity and with different degrees of glucose impairment
Source: Sci Rep. 2019 Aug 5;9:11333. doi: 10.1038/s41598-019-47719-y (PMC6683173; doi:10.1038/s41598-019-47719-y)
Supplement: Supplementary file 1 — Characterization of subcutaneous and omental adipose tissue in patients with obesity and with different degrees of glucose impairment [file 41598_2019_47719_MOESM1_ESM.pdf]

**Supplementary Information**

# Characterization of subcutaneous and omental adipose tissue in patients with obesity and with different degrees of glucose impairment

Anna Belligoli[1,2,#], Chiara Compagnin[1,2,#], Marta Sanna[1,2], Francesca Favaretto[1,2], Roberto Fabris[1,2], Luca Busetto[1,2], Mirto Foletto[2], Chiara Dal Prà[1,2], Roberto Serra[1,2], Luca Prevedello[2], Chiara Da Re[3], Romeo Bardini[3], Claudia Mescoli[4], Massimo Rugge[4], Paola Fioretto[1], Scilla Conci[1,2], Silvia Bettini[1,2], Gabriella Milan[1,2,*] & Roberto Vettor[1,2]

[1]Department of Medicine, University of Padua, Internal Medicine 3, 35128 Padua, Italy.

[2]Center for the Study and the Integrated Treatment of Obesity, Padua Hospital, 35128 Padua, Italy.

[3]Department of Surgical, Oncological and Gastroenterological Sciences, University of Padua, Division of General Surgery, 35128 Padua, Italy.

[4]Department of Medicine, University of Padua, Surgical Pathology and Cytopathology Unit, 35121 Padua, Italy.

**Additional footnotes**

[#]These authors contributed equally

[*]Corresponding Author

**Corresponding author**

Dr. Gabriella Milan, B.D., Ph.D.

Department of Medicine

University of Padua

via Ospedale, 105

35128 Padova, Italy

Phone +39-049-8218550;

Fax +39-049-8218555

E-mail: gabriella.milan@unipd.it

## Supplementary Tables, Figures and Legends

| | **Patient Groups** | | | | ***P* value** | | | | | |
|---|---|---|---|---|---|---|---|---|---|---|
| | lean (n=10) | ob N (n=5) | ob preDM (n=5) | ob T2DM (n=5) | lean vs. ob N | lean vs. ob preDM | lean vs. ob T2DM | ob N vs. ob preDM | ob N vs. ob T2DM | ob preDM vs. ob T2DM |
| Female sex-n° (%) | 6(60) | 4(80) | 4(80) | 2(40) | 0.6 | 0.6 | 0.61 | 1.0 | 0.52 | 0.52 |
| Age (y) | 52±12 | 42±9 | 51±11 | 44±13 | 0.36 | 0.36 | 0.36 | 0.36 | 0.36 | 0.36 |
| Hypertension-n° (%) | 2(20) | 1(20) | 3(60) | 4(80) | 1.0 | 0.25 | 0.09 | 0.52 | 0.21 | 1.0 |
| Dyslipidemia- n° (%) | 0(0) | 3(60) | 4(80) | 5(100) | 0.02 | 0.004 | <0.001 | 1.0 | 0.44 | 1.0 |
| OSAS- n° (%) | 0(0) | 1(20) | 3(60) | 1(20) | 0.33 | 0.02 | 0.33 | 0.52 | 1.0 | 0.52 |
| BMI (kg/m$^2$) * | 24±2 | 54±19 | 47±5 | 57±9 | <0.001 | 0.007 | <0.001 | 0.58 | 0.63 | 0.41 |
| FPG (mmol/L) | 5.2±0.6 | 4.9±0.2 | 5.4±0.6 | 10.0±4.3 | 1.0 | 1.0 | 0.02 | 1.0 | 0.006 | 0.16 |
| Insulin (mU/L) | - | 15±9 | 16±9 | 45±22 | - | - | - | 0.97 | 0.03 | 0.02 |
| $HOMA_{IR}$ † | - | 3.3±1.9 | 3.6±1.8 | 17±9 | - | - | - | 1.0 | 0.03 | 0.05 |
| Glucose-AUC (mmol/L)min ‡ | - | 1138±148 | 1451±132 | - | - | - | - | 0.008 | - | - |
| Insulin-AUC (mU/L)min ‡ | - | 10535±4853 | 15463±12065 | - | - | - | - | 0.84 | - | - |
| Waist (cm) § | - | 127±19 | 132±16 | 154±16 | - | - | - | 0.10 | 0.10 | 0.10 |
| Total Cholesterol (mg/dL) | - | 193±27 | 213±47 | 225±47 | - | - | - | 0.48 | 0.48 | 0.48 |
| HDL-Cholesterol (mg/dL) | - | 51±9 | 49±15 | 41±8 | - | - | - | 0.33 | 0.33 | 0.33 |
| LDL-Cholesterol (mg/dL) | - | 123±17 | 140±28 | 154±38 | - | - | - | 0.28 | 0.28 | 0.28 |
| Triglycerides (mg/dL) | - | 93±26 | 107±42 | 223±77 | - | - | - | 0.67 | 0.006 | 0.009 |
| hs-CRP (mg/L) | - | 4.3±2.6 | 5.5±3.7 | 9.3±1.7 | - | - | - | 0.51 | 0.04 | 0.12 |
| TNF-α (ng/L) | - | 8.4±2.8 | 10±4.5 | 10.7±2.9 | - | - | - | 0.57 | 0.57 | 0.57 |
| IL-6 (ng/L) | - | 3.2±1.5 | 3.1±1.6 | 4.5±2.3 | - | - | - | 0.44 | 0.44 | 0.44 |
| Leptin (μg/L) | - | 33±17 | 43±10 | 38±18 | - | - | - | 0.68 | 0.68 | 0.68 |

**Supplementary Table S1. Characteristics and Clinical Parameters of the Lean Subjects and Obese Patients analyzed by IHC.** Clinical data of control subjects (lean), normoglycemic (ob N), prediabetic (ob preDM) and diabetic (ob T2DM) obese patients are presented as mean values ± SD. Statistical analysis was based on the One Way ANOVA test (age, BMI, insulin, waist, total cholesterol, HDL- and LDL-cholesterol, triglycerides, hs-CRP, TNF-α, IL-6, leptin) or a non-parametric Kruskal-Wallis test (FPG, $HOMA_{IR}$) followed, when statistically significant by, respectively, the Holm-Sidak test or Dunn's multiple comparison post-test. The unpaired t-test and Wilcoxon Mann Whitney test was used to compare respectively glucose- and insulin-AUC values. Pearson's chi-square test or Fisher's exact test were used to compare dichotomous variables between groups.

[*]BMI was calculated by dividing weight in kilograms by the square of height in meters. [†]$HOMA_{IR}$ was calculated according to the following formula: [fasting serum insulin (mU/L) x fasting plasma glucose (mmol/L)]/22.5. [‡]AUC was calculated using the trapezoidal method. [§]Waist circumference was measured with a tailor-meter at the midpoint of the line connecting the inferior border of the lowest rib with the superior border of the iliac crest.

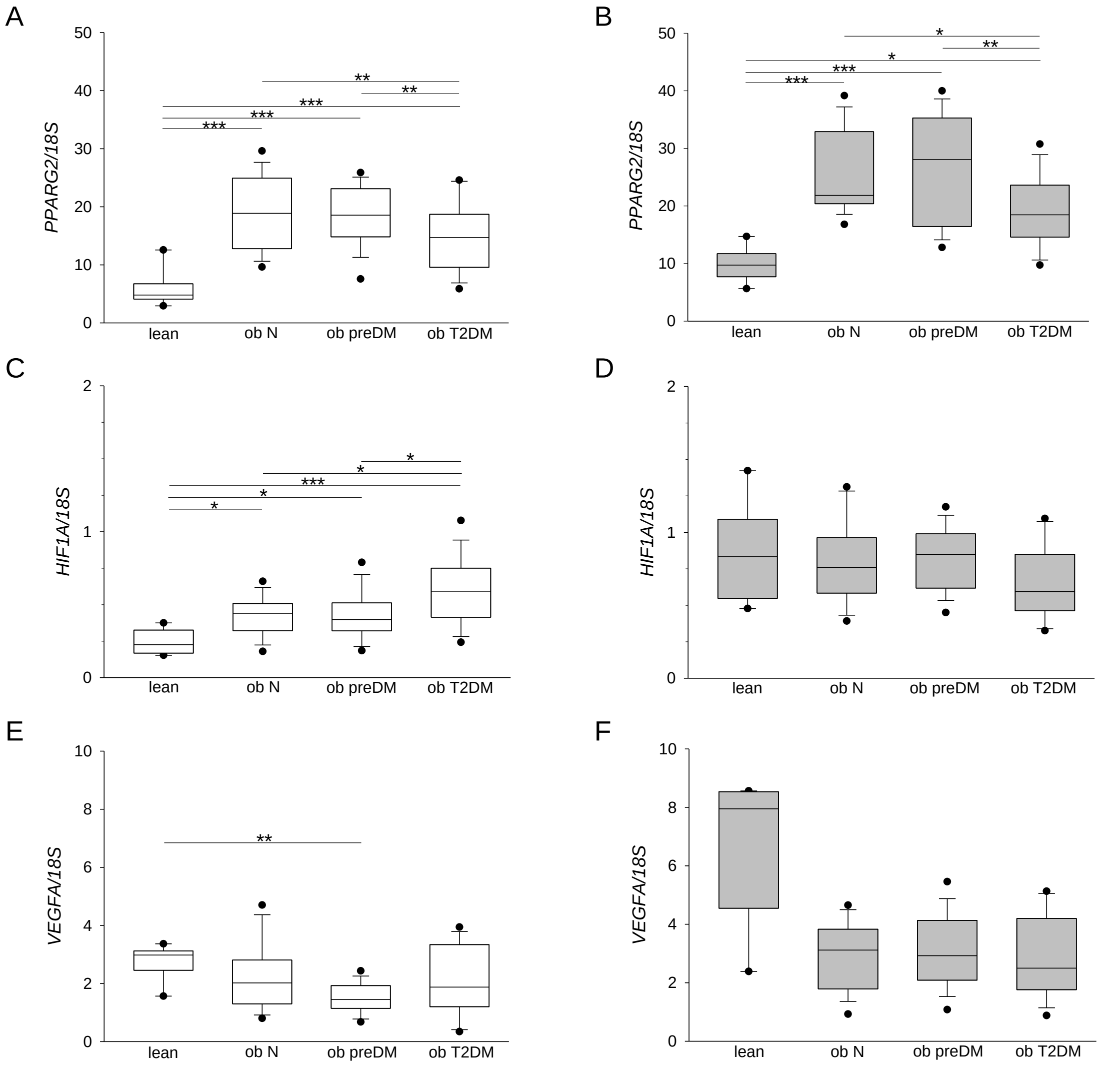


**Supplementary Figure S1. *PPARG2*, *HIF1A* and *VEGFA* expression in human SAT and VAT.** *PPARG2* (**A**,**B**), *HIF1A* (**C**,**D**), *VEGFA* (**E**,**F**) mRNA levels in SAT (white boxes, **A,C,E**) and VAT (grey boxes, **B,D,F**), normalized to *18S* rRNA content, are plotted for lean subjects, normoglycemic (ob N), prediabetic (ob preDM) and diabetic (ob T2DM) obese patients.

Data are reported as box plot graph with the lowest and highest values (wiskers), the medians (lines) and 5$^{th}$ and 95$^{th}$ percentiles (black circles outside the whiskers). Statistical analysis was performed using One Way ANOVA test followed by Holm-Sidak post-test in (**A**) and using Kruskal-Wallis test followed by Dunn's multiple comparison post-hoc test in (**B**-**F**), $*P \leq 0.05$, $**P \leq 0.01$, $***P \leq 0.001$. The patients analyzed were: 5 lean, 19 ob N, 19 ob preDM, 18 ob T2DM in (**A**,**C**,**E**) and 4 lean, 17 ob N, 21 ob preDM, 20 ob T2DM in (**B**,**D**,**F**).

| | Patient Groups | | | | *P* value | | | | | |
|---|---|---|---|---|---|---|---|---|---|---|
| | lean (n=6) | ob N (n=5) | ob preDM (n=5) | ob T2DM (n=5) | lean vs. ob N | lean vs. ob preDM | lean vs. ob T2DM | ob N vs. ob preDM | ob N vs. ob T2DM | ob preDM vs. ob T2DM |
| Female sex-n° (%) | 6(100) | 5(100) | 4(80) | 3(60) | 1.0 | 0.46 | 0.18 | 1.0 | 0.44 | 1.0 |
| Age (y) | 47±12 | 35±7 | 43±12 | 51±9 | 0.13 | 0.13 | 0.13 | 0.13 | 0.13 | 0.13 |
| Hypertension-n° (%) | 0(0) | 2(40) | 3(60) | 5(100) | 0.18 | 0.06 | 0.002 | 1.0 | 0.17 | 0.44 |
| Dyslipidemia- n° (%) | 0(0) | 4(80) | 2(40) | 3(60) | 0.15 | 0.18 | 0.06 | 0.52 | 1.0 | 1.0 |
| OSAS- n° (%) | 0(0) | 0(0) | 1(20) | 1(20) | 1.0 | 0.46 | 0.46 | 1.0 | 1.0 | 1. 0 |
| BMI (kg/m$^2$) * | 21±1 | 46±6 | 52±7 | 46±6 | <0.001 | <0.001 | <0.001 | 0.24 | 0.88 | 0.27 |
| FPG (mmol/L) | 4.8±0.4 | 4.9±0.4 | 5.8±0.5 | 9.4±3.3 | 1.0 | 0.21 | 0.002 | 0.50 | 0.01 | 1.0 |
| Insulin (mU/L) | - | 12±7 | 27±14 | 24±11 | - | - | - | 0.15 | 0.15 | 0.15 |
| $HOMA_{IR}$ † | - | 2.7±1.8 | 7.1±4.2 | 9.7±4.1 | - | - | - | 0.14 | 0.04 | 0.28 |
| Glucose-AUC (mmol/L)min ‡ | - | 1189±69 | 1524±100 | - | - | - | - | 0.008 | - | - |
| Insulin-AUC (mU/L)min ‡ | - | 12465±5126 | 15426±4876 | - | - | - | - | 0.38 | - | - |
| Waist (cm) § | - | 126±15 | 131±13 | 134±17 | - | - | - | 0.78 | 0.78 | 0.78 |
| Total Cholesterol (mg/dL) | - | 205±36 | 186±27 | 164±22 | - | - | - | 0.12 | 0.12 | 0.12 |
| HDL-Cholesterol (mg/dL) | - | 60±18 | 40±6 | 46±7 | - | - | - | 0.06 | 0.06 | 0.06 |
| LDL-Cholesterol (mg/dL) | - | 123±27 | 130±38 | 93±22 | - | - | - | 0.16 | 0.16 | 0.16 |
| Triglycerides (mg/dL) | - | 113±56 | 122±45 | 126±41 | - | - | - | 0.92 | 0.92 | 0.92 |
| hs-CRP (mg/L) | - | 5.4±4.5 | 9.0±3.7 | 6.5±3.7 | - | - | - | 0.38 | 0.38 | 0.38 |
| TNF-α (ng/L) | - | 5.9±0.4 | 6.4±1.3 | 7.1±3.0 | - | - | - | 0.48 | 0.48 | 0.48 |
| IL-6 (ng/L) | - | 5.3±4.0 | 2.8±0.7 | 4.6±1.5 | - | - | - | 0.30 | 0.30 | 0.30 |
| Leptin (μg/L) | - | 53±13 | 40±7 | 31±12 | - | - | - | 0.15 | 0.03 | 0.25 |

**Supplementary Table S2. Characteristics and Clinical Parameters of the Lean Subjects and Obese Patients analyzed by TEM.** Clinical data of control subjects (lean), normoglycemic (ob N), prediabetic (ob preDM) and diabetic (ob T2DM) obese patients are presented as mean values ± SD. Statistical analysis was based on the One Way ANOVA test (age, BMI, insulin, $HOMA_{IR}$, waist, total cholesterol, HDL- and LDL-cholesterol, triglycerides, hs-CRP, IL-6, leptin) or a non-parametric Kruskal-Wallis test (FPG, TNF-α) followed, when statistically significant by, respectively, the Holm-Sidak test or Dunn's multiple comparison post-test. The Wilcoxon Mann Whitney test and unpaired t-test was used to compare respectively glucose- and insulin-AUC values. Pearson's chi-square test or Fisher's exact test were used to compare dichotomous variables between groups.

[*]BMI was calculated by dividing weight in kilograms by the square of height in meters. [†]$HOMA_{IR}$ was calculated according to the following formula: [fasting serum insulin (mU/L) x fasting plasma glucose (mmol/L)]/22.5. [‡]AUC was calculated using the trapezoidal method. [§]Waist circumference was measured with a tailor-meter at the midpoint of the line connecting the inferior border of the lowest rib with the superior border of the iliac crest.

| | Patient Groups | | | | *P* value | | | | | |
|---|---|---|---|---|---|---|---|---|---|---|
| | lean (n=4) | ob N (n=20) | ob preDM (n=15) | ob T2DM (n=23) | lean vs. ob N | lean vs. ob preDM | lean vs. ob T2DM | ob N vs. ob preDM | ob N vs. ob T2DM | ob preDM vs. ob T2DM |
| Female sex-n° (%) | 2(50) | 15(75) | 13(87) | 14(61) | 0.55 | 0.18 | 1.0 | 0.67 | 0.51 | 0.15 |
| Age (y) | 47±14 | 43±10 | 45±9 | 51±11 | 0.05 | 0.05 | 0.05 | 0.05 | 0.05 | 0.05 |
| Hypertension-n° (%) | 1(25) | 12(60) | 7(47) | 20(87) | 0.3 | 0.60 | 0.03 | 0.66 | 0.10 | 0.01 |
| Dyslipidemia- n° (%) | 0(0) | 12(60) | 10(67) | 21(91) | 0.09 | 0.03 | <0.001 | 0.96 | 0.03 | 0.09 |
| OSAS- n° (%) | 0(0) | 4(20) | 6(40) | 11(48) | 1.0 | 0.25 | 0.12 | 0.27 | 0.11 | 0.89 |
| BMI ($kg/m^2$) * | 24±2 | 49±12 | 49±7 | 49±9 | 0.06 | 0.03 | 0.02 | 1.0 | 1.0 | 1.0 |
| FPG (mmol/L) | 5.4±0.9 | 5.0±0.4 | 5.8±0.7 | 8.8±2.8 | 1.0 | 1.0 | 0.02 | 0.12 | <0.001 | 0.005 |
| Insulin (mU/L) | - | 18±13 | 32±27 | 32±18 | - | - | - | 0.13 | 0.03 | 1.0 |
| $HOMA_{IR}$ † | - | 3.9±3.0 | 8.4±8.3 | 12±7 | - | - | - | 0.08 | <0.001 | 0.44 |
| Glucose-AUC (mmol/L)min ‡ | - | 1158±139 | 1489±162 | - | - | - | - | <0.001 | - | - |
| Insulin-AUC (mU/L)min ‡ | - | 11916±6291 | 18788±12589 | - | - | - | - | 0.12 | - | - |
| Waist (cm) § | - | 131±16 | 137±12 | 138±17 | - | - | - | 0.34 | 0.34 | 0.34 |
| Total Cholesterol (mg/dL) | - | 188±28 | 205±35 | 194±46 | - | - | - | 0.41 | 0.41 | 0.41 |
| HDL-Cholesterol (mg/dL) | - | 46±10 | 50±12 | 41±11 | - | - | - | 0.09 | 0.09 | 0.09 |
| LDL-Cholesterol (mg/dL) | - | 121±25 | 129±34 | 123±38 | - | - | - | 0.84 | 0.84 | 0.84 |
| Triglycerides (mg/dL) | - | 119±43 | 154±93 | 174±73 | - | - | - | 1.0 | 0.04 | 0.55 |
| hs-CRP (mg/L) | - | 5.5±3.7 | 7.3±3.5 | 7.7±2.8 | - | - | - | 0.17 | 0.17 | 0.17 |
| TNF-α (ng/L) | - | 8.3±2.1 | 8.5±2.9 | 10.3±3.1 | - | - | - | 0.08 | 0.08 | 0.08 |
| IL-6 (ng/L) | - | 3.6±1.6 | 3.8±1.5 | 4.2±2.6 | - | - | - | 0.91 | 0.91 | 0.91 |
| Leptin (μg/L) | - | 38±16 | 44±13 | 38±15 | - | - | - | 0.46 | 0.46 | 0.46 |

**Supplementary Table S3. Characteristics and Clinical Parameters of the Lean Subjects and Obese Patients analyzed by flow cytometry using SAT biopsies.** Clinical data of control subjects (lean), normoglycemic (ob N), prediabetic (ob preDM) and diabetic (ob T2DM) obese patients are presented as mean values ± SD. Statistical analysis was based on the One Way ANOVA test (age, waist, total cholesterol, HDL- and LDL-cholesterol, leptin) or a non-parametric Kruskal-Wallis test (BMI, FPG, insulin, $HOMA_{IR}$, triglycerides, hs-CRP, TNF-α, IL-6) followed, when statistically significant by, respectively, the Holm-Sidak test or Dunn's multiple comparison post-test. The unpaired t-test and Wilcoxon Mann Whitney test was used to compare respectively glucose- and insulin-AUC values. Pearson's chi-square test or Fisher's exact test were used to compare dichotomous variables between groups.

[*]BMI was calculated by dividing weight in kilograms by the square of height in meters. [†]$HOMA_{IR}$ was calculated according to the following formula: [fasting serum insulin (mU/L) x fasting plasma glucose (mmol/L)]/22.5. [‡]AUC was calculated using the trapezoidal method. [§]Waist circumference was measured with a tailor-meter at the midpoint of the line connecting the inferior border of the lowest rib with the superior border of the iliac crest.

| | Patient Groups | | | | *P* value | | | | | |
|---|---|---|---|---|---|---|---|---|---|---|
| | lean<br><br>(n=9) | ob N<br><br>(n=23) | ob preDM<br><br>(n=21) | ob T2DM<br><br>(n=30) | lean<br>vs.<br>ob N | lean<br>vs.<br>ob preDM | lean<br>vs.<br>ob T2DM | ob N<br>vs.<br>ob preDM | ob N<br>vs.<br>ob T2DM | ob preDM<br>vs.<br>ob T2DM |
| Female sex-n° (%) | 4(44) | 18(78) | 17(81) | 17(57) | 0.10 | 0.08 | 0.71 | 1.0 | 0.18 | 0.13 |
| Age (y) | 49±12 | 41±10 | 44±9 | 51±10 | 0.17 | 0.50 | 0.52 | 0.56 | 0.002 | 0.05 |
| Hypertension-n° (%) | 1(11) | 10(43) | 12(57) | 27(90) | 0.12 | 0.04 | <0.001 | 0.55 | <0.001 | 0.02 |
| Dyslipidemia- n° (%) | 0(0) | 12(52) | 11(52) | 25(83) | 0.01 | 0.01 | <0.001 | 0.77 | 0.03 | 0.04 |
| OSAS- n° (%) | 0(0) | 4(17) | 5(24) | 13(43) | 0.30 | 0.29 | 0.02 | 0.72 | 0.09 | 0.26 |
| BMI (kg/m$^2$) * | 24±2 | 47±9 | 50±9 | 49±9 | 0.004 | <0.001 | <0.001 | 0.79 | 1.0 | 1.0 |
| FPG (mmol/L) | 5.2±0.6 | 5.0±0.4 | 5.8±0.7 | 9.8±4.4 | 1.0 | 0.66 | <0.001 | 0.07 | <0.001 | <0.001 |
| Insulin (mU/L) | - | 17±12 | 32±25 | 41±42 | - | - | - | 0.02 | 0.004 | 1.0 |
| $HOMA_{IR}$ † | - | 3.9±2.7 | 8.5±7.1 | 16±16 | - | - | - | 0.009 | <0.001 | 0.31 |
| Glucose-AUC (mmol/L)min ‡ | - | 1152±134 | 1444±151 | - | - | - | - | <0.001 | - | - |
| Insulin-AUC (mU/L)min ‡ | - | 11299±5890 | 19192±12247 | - | - | - | - | 0.02 | - | - |
| Waist (cm) § | - | 129±15 | 137±11 | 137±16 | - | - | - | 0.11 | 0.11 | 0.11 |
| Total Cholesterol (mg/dL) | - | 184±28 | 197±43 | 193±43 | - | - | - | 0.54 | 0.54 | 0.54 |
| HDL-Cholesterol (mg/dL) | - | 46±9 | 48±13 | 41±12 | - | - | - | 0.13 | 0.13 | 0.13 |
| LDL-Cholesterol (mg/dL) | - | 118±25 | 124±47 | 123±37 | - | - | - | 0.81 | 0.81 | 0.81 |
| Triglycerides (mg/dL) | - | 116±43 | 158±108 | 178±74 | - | - | - | 0.95 | 0.006 | 0.16 |
| hs-CRP (mg/L) | - | 5.4±3.8 | 7.0±3.6 | 7.6±3.0 | - | - | - | 0.10 | 0.10 | 0.10 |
| TNF-α (ng/L) | - | 8.7±3.5 | 8.7±3.7 | 9.7±2.8 | - | - | - | 0.20 | 0.20 | 0.20 |
| IL-6 (ng/L) | - | 3.8±3.0 | 3.5±1.3 | 4.4±2.6 | - | - | - | 0.57 | 0.57 | 0.57 |
| Leptin (µg/L) | - | 38±16 | 45±18 | 37±15 | - | - | - | 0.25 | 0.25 | 0.25 |

**Supplementary Table S4. Characteristics and Clinical Parameters of the Lean Subjects and Obese Patients analyzed by flow cytometry using VAT biopsies.** Clinical data of control subjects (lean), normoglycemic (ob N), prediabetic (ob preDM) and diabetic (ob T2DM) obese patients are presented as mean values ± SD. Statistical analysis was based on the One Way ANOVA test (age, waist, total cholesterol, HDL-cholesterol, leptin) or a non-parametric Kruskal-Wallis test (BMI, FPG, insulin, $HOMA_{IR}$, LDL-cholesterol, triglycerides, hs-CRP, TNF-α, IL-6) followed, when statistically significant by, respectively, the Holm-Sidak test or Dunn's multiple comparison post-test. The unpaired t-test was used to compare glucose- and insulin-AUC values. Pearson's chi-square test or Fisher's exact test were used to compare dichotomous variables between groups.

[*]BMI was calculated by dividing weight in kilograms by the square of height in meters. [†]$HOMA_{IR}$ was calculated according to the following formula: [fasting serum insulin (mU/L) x fasting plasma glucose (mmol/L)]/22.5. [‡]AUC was calculated using the trapezoidal method. [§]Waist circumference was measured with a tailor-meter at the midpoint of the line connecting the inferior border of the lowest rib with the superior border of the iliac crest.

| | Patient Groups | | | P value | | |
|---|---|---|---|---|---|---|
| | ob N<br><br>(n=18) | ob preDM<br><br>(n=15) | ob T2DM<br><br>(n=13) | ob N<br>vs.<br>ob preDM | ob N<br>vs.<br>ob T2DM | ob preDM<br>vs.<br>ob T2DM |
| Female sex-n° (%) | 15(83) | 13(87) | 10(77) | 1.0 | 0.68 | 0.64 |
| Age (y) | 38±11 | 46±10 | 48±11 | 0.07 | 0.04 | 0.62 |
| Hypertension-n° (%) | 6(33) | 6(40) | 12(92) | 0.97 | 0.004 | 0.006 |
| Dyslipidemia- n° (%) | 11(61) | 8(53) | 11(85) | 0.93 | 0.24 | 0.11 |
| OSAS- n° (%) | 2(11) | 4(27) | 5(38) | 0.38 | 0.10 | 0.69 |
| BMI (kg/m$^2$) * | 49±12 | 50±9 | 51±7 | 0.51 | 0.51 | 0.51 |
| FPG (mmol/L) | 4.9±0.3 | 5.8±0.7 | 12.1±5.5 | 0.02 | <0.001 | 0.007 |
| Insulin (mU/L) | 19±12 | 36±26 | 57±63 | 0.01 | 0.008 | 1.0 |
| $HOMA_{IR}$ † | 4.1±2.9 | 9.6±7.6 | 23±24 | 0.007 | <0.001 | 0.44 |
| Glucose-AUC (mmol/L)min ‡ | 1174±147 | 1529±194 | - | <0.001 | - | - |
| Insulin-AUC (mU/L)min ‡ | 12962±6034 | 22513±11685 | - | 0.01 | - | - |
| Waist (cm) § | 129±15 | 135±14 | 140±13 | 0.09 | 0.09 | 0.09 |
| Total Cholesterol (mg/dL) | 187±30 | 197±27 | 179±40 | 0.36 | 0.36 | 0.36 |
| HDL-Cholesterol (mg/dL) | 45±13 | 48±11 | 41±14 | 0.42 | 0.42 | 0.42 |
| LDL-Cholesterol (mg/dL) | 119±30 | 121±31 | 110±33 | 0.66 | 0.66 | 0.66 |
| Triglycerides (mg/dL) | 134±77 | 155±80 | 185±86 | 0.14 | 0.14 | 0.14 |
| hs-CRP (mg/L) | 4.9±3.7 | 7.5±3.4 | 7.8±2.9 | 0.06 | 0.06 | 0.06 |
| TNF-α (ng/L) | 8.3±2.8 | 9.0±3.7 | 9.7±3.1 | 0.42 | 0.42 | 0.42 |
| IL-6 (ng/L) | 2.9±1.0 | 4.0±1.2 | 5.0±2.9 | 0.03 | 0.05 | 1.0 |
| Leptin (μg/L) | 40±17 | 46±17 | 39±16 | 0.50 | 0.50 | 0.50 |

**Supplementary Table S5. Characteristics and Clinical Parameters of the Obese Patients analyzed by *in vitro* adipogenic culturing from SAT biopsies.** Clinical data of normoglycemic (ob N), prediabetic (ob preDM) and diabetic (ob T2DM) obese patients are presented as mean values ± SD. Statistical analysis was based on the One Way ANOVA test (age, waist, total cholesterol, HDL- and LDL-cholesterol, leptin) or a non-parametric Kruskal-Wallis test (BMI, FPG, insulin, $HOMA_{IR}$, triglycerides, hs-CRP, TNF-α, IL-6) followed, when statistically significant by, respectively, the Holm-Sidak test or Dunn's multiple comparison post-test. The unpaired t-test and Wilcoxon Mann Whitney test was used to compare respectively glucose- and insulin-AUC values. Pearson's chi-square test or Fisher's exact test were used to compare dichotomous variables between groups.
[*]BMI was calculated by dividing weight in kilograms by the square of height in meters. [†]$HOMA_{IR}$ was calculated according to the following formula: [fasting serum insulin (mU/L) x fasting plasma glucose (mmol/L)]/22.5. [‡]AUC was calculated using the trapezoidal method. [§]Waist circumference was measured with a tailor-meter at the midpoint of the line connecting the inferior border of the lowest rib with the superior border of the iliac crest.

| | Patient Groups | | | P value | | |
|---|---|---|---|---|---|---|
| | ob N<br><br>(n=20) | ob preDM<br><br>(n=25) | ob T2DM<br><br>(n=23) | ob N<br>vs.<br>ob preDM | ob N<br>vs.<br>ob T2DM | ob preDM<br>vs.<br>ob T2DM |
| Female sex-n° (%) | 17(85) | 20(80) | 13(57) | 0.72 | 0.09 | 0.15 |
| Age (y) | 42±8 | 46±10 | 52±10 | 0.19 | 0.005 | 0.08 |
| Hypertension-n° (%) | 7(35) | 12(48) | 21(91) | 0.57 | <0.001 | 0.003 |
| Dyslipidemia- n° (%) | 11(55) | 14(56) | 20(87) | 0.81 | 0.05 | 0.04 |
| OSAS- n° (%) | 4(20) | 8(32) | 10(43) | 0.57 | 0.19 | 0.60 |
| BMI (kg/m²) * | 49±11 | 48±8 | 49±9 | 0.78 | 0.78 | 0.78 |
| FPG (mmol/L) | 5.0±0.2 | 5.8±0.6 | 10.3±4.8 | 0.014 | <0.001 | <0.001 |
| Insulin (mU/L) | 17±11 | 31±25 | 45±45 | 0.04 | 0.004 | 0.92 |
| $HOMA_{IR}$ † | 3.9±2.8 | 8.2±7.1 | 17±18 | 0.02 | <0.001 | 0.13 |
| Glucose-AUC (mmol/L)min ‡ | 1156±132 | 1510±177 | - | <0.001 | - | - |
| Insulin-AUC (mU/L)min ‡ | 12029±6169 | 20095±12337 | - | 0.03 | - | - |
| Waist (cm) § | 128±15 | 134±12 | 137±18 | 0.15 | 0.15 | 0.15 |
| Total Cholesterol (mg/dL) | 191±28 | 202±41 | 194±47 | 0.65 | 0.65 | 0.65 |
| HDL-Cholesterol (mg/dL) | 48±9 | 49±12 | 41±12 | 0.76 | 0.09 | 0.06 |
| LDL-Cholesterol (mg/dL) | 123±24 | 127±46 | 123±40 | 0.99 | 0.99 | 0.99 |
| Triglycerides (mg/dL) | 116±46 | 160±100 | 176±82 | 0.55 | 0.03 | 0.56 |
| hs-CRP (mg/L) | 5.1±3.5 | 6.8±3.6 | 8.0±2.8 | 0.34 | 0.03 | 0.89 |
| TNF-α (ng/L) | 7.7±2.5 | 8.3±3.2 | 9.7±3.1 | 0.09 | 0.09 | 0.09 |
| IL-6 (ng/L) | 3.0±1.1 | 3.5±1.2 | 4.6±2.5 | 0.08 | 0.08 | 0.08 |
| Leptin (µg/L) | 41±17 | 42±17 | 38±16 | 0.71 | 0.71 | 0.71 |

**Supplementary Table S6. Characteristics and Clinical Parameters of the Obese Patients analyzed by *in vitro* adipogenic culturing from VAT biopsies.** Clinical data of normoglycemic (ob N), prediabetic (ob preDM) and diabetic (ob T2DM) obese patients are presented as mean values ± SD. Statistical analysis was based on the One Way ANOVA test (age, waist, total cholesterol, HDL-cholesterol, leptin) or a non-parametric Kruskal-Wallis test (BMI, FPG, insulin, $HOMA_{IR}$, LDL-cholesterol, triglycerides, hs-CRP, TNF-α, IL-6) followed, when statistically significant by, respectively, the Holm-Sidak test or Dunn's multiple comparison post-test. The unpaired t-test and Wilcoxon Mann Whitney test was used to compare respectively glucose- and insulin-AUC values. Pearson's chi-square test or Fisher's exact test were used to compare dichotomous variables between groups.

[*]BMI was calculated by dividing weight in kilograms by the square of height in meters. [†]$HOMA_{IR}$ was calculated according to the following formula: [fasting serum insulin (mU/L) x fasting plasma glucose (mmol/L)]/22.5. [‡]AUC was calculated using the trapezoidal method. [§]Waist circumference was measured with a tailor-meter at the midpoint of the line connecting the inferior border of the lowest rib with the superior border of the iliac crest.

| | | Patient groups | | | |
|---|---|---|---|---|---|
| | | **lean** | **ob N** | **ob preDM** | **ob T2DM** |
| **SAT** | **CD73+** | 90.0 (87.0-91.7) | 97.8 (94.4-98.9) | 96.3 (88.7-97.8) | 92.9 (92.2-94.5) |
| | **CD90+** | 92.6 (90.0-95.3) | 97.7 (96.5-99.1) | 99.0 (96.1-99.6) | 99.3 (95.0-99.7) |
| | **CD105+** | 42.1 (24.9-59.4) | 51.9 (25.3-59.9) | 42.0 (17.3-49.6) | 30.3 (21.9-52.2) |
| | **CD271+** | 27.2 | 37.3 (22.6-44.6) | 42.3 (24.2-44.4) | 31.9 (26.4-45.5) |
| | **CD146+** | 1.0 | 0.5 (0.3-0.9) | 0.5 (0.2-0.8) | 0.6 (0.4-0.9) |
| **VAT** | **CD73+** | 97.1 (95.3-97.5) | 94.3 (86.5-98.4) | 93.8 (85.9-96.9) | 94.2 (92.4-95.5) |
| | **CD90+** | 92.8 (82.6-98.3) | 95.6 (91.3-97.8) | 95.2 (90.2-98.6) | 95.8 (93.3-98.4) |
| | **CD105+** | 47.8 (33.6-59.8) | 63.7 (59.1-75.6)* | 63.9 (53.9-77.5)** | 67.8 (61.1-72.2)** |
| | **CD271+** | 42.4 (28.4-46.5) | 70.5 (56.9-78.8)*** | 68.2 (47.4-83.4)*** | 59.9 (55.0-71.5)** |
| | **CD146+** | 0.6 (0.03-1.3) | 1.0 (0.4-1.9) | 0.6 (0.2-1.5) | 1.3 (0.5-1.8) |

**Supplementary Table S7. Quantification by flow cytometry of CD73+, CD90+, CD105+, CD271+ and CD146+ cells in the CD45-/31-/34+ subpopulation of human SAT and VAT in the lean controls and obese patients.** Percentages of CD45-/31-/34+ cells expressing CD73, CD90, CD105, CD271 and CD146 antigens in SAT- and VAT-SVFs of lean control subjects, normoglycemic (ob N), prediabetic (ob preDM) and diabetic (ob T2DM) obese patients. Data are reported as median percentage ($25^{th}$-$75^{th}$ percentile). The statistical analysis was based on One Way ANOVA (Fisher LSD method) and Kruskal-Wallis test (Dunn's method); ob *vs* lean: *$P \leq 0.05$, **$P \leq 0.01$, ***$P \leq 0.001$.

| | FITC | PE | PerCP-Cy5.5 | APC |
|---|---|---|---|---|
| **Unstained** | / | / | / | / |
| **Negative** | IgG1 | IgG1 | IgG1 | IgG1 |
| **Sample 1** | CD45 | CD31 | CD34 | / |
| **Sample 2** | CD45 CD31 | CD90 | CD34 | CD73 |
| **Sample 3** | CD45 CD31 | CD146 | CD34 | CD271 |
| **Sample 4** | CD45 CD31 | / | CD34 | CD105 |

**Supplementary Table S8. Antibody flow cytometry panel for immunophenotyping of SVF cells.** SVF cells isolated from AT of the patients analyzed were incubated with a combination of monoclonal mouse anti-human fluorochrome-conjugated antibodies specific for the different antigens in order to perform multiparametric flow cytometry analysis. Isotype-matched FITC-, PE-, PerCP-Cy5.5- and APC-IgG1 monoclonal antibodies were used as a negative control. Unstained sample was used for auto-fluorescence control.

| GENE | FORWARD (5'-3') | REVERSE (5'-3') | PRIMER F/R (nM) | ANNEALING (°C) | AMPLICON SIZE (bp) |
|---|---|---|---|---|---|
| ***RNA18S*** | CGGCTACCACATCCAAGGAA | GCTGGAATTACCGCGGCT | 100/100 | 60 | 186 |
| ***HIF1A*** | TTACCATGCCCCAGATTCAG | GGTTCTTTGCTTCTGTGTCTTC | 300/300 | 58 | 180 |
| ***VEGFA*** | TCACCATGCAGATTATGCGGA | TGTTGTGCTGTAGGAAGCTCA | 300/300 | 58 | 75 |
| ***PPARG2*** | ACCCAGAAAGCGATTCCTTCA | AGTGGTCTTCCATTACGGAGAGATC | 900/900 | 60 | 87 |
| ***FABP4*** | GAAGTCAAGAGCACCATAACC | CCACCACCAGTTTATCATCC | 300/300 | 53 | 113 |
| ***ADIPOQ*** | ACAATGGCACACCAGGCCGTGA | AGCGGCTTCTCCAGGCTCTCCTTT | 300/300 | 58 | 179 |

**Supplementary Table S9. Real Time PCR conditions.** Primer sequences and concentrations, annealing temperatures and amplicon sizes are outlined for each mRNA quantified by real time PCR. *RNA18S* = 18S ribosomal RNA; *HIF1A* = hypoxia inducible factor 1 alpha subunit; *VEGFA* = vascular endothelial growth factor A; *PPARG2* = peroxisome proliferator activated receptor gamma isoform 2; *FABP4* = fatty acid binding protein 4; *ADIPOQ* = adiponectin, C1Q and collagen domain containing.
